# Effect of button layout on the exploration and learning of robot operation using an unfamiliar controller

**DOI:** 10.1371/journal.pone.0272782

**Published:** 2022-09-02

**Authors:** Tetsuyou Watanabe

**Affiliations:** Faculty of Frontier Engineering, Kanazawa University, Kanazawa, Ichikawa, Japan; Dai Hoc Duy Tan, VIET NAM

## Abstract

Robots are becoming increasingly accessible to both experts and non-experts. Therefore, establishing a method for learning robot operations that can be easily mastered by non-experts is important. With this in mind, we aimed to develop a method that facilitates skill acquisition for non-experts that operate robots. As a first step, this study examined the effects of button layout on the exploration and learning of robot operations. A humanoid robot was operated using an unfamiliar tablet-based user interface to achieve the task of shifting the robot’s posture to the desired posture: single-foot-standing. The process in which participants found and repeated sequences of commands to achieve the shift task was observed. Four types of button layouts were prepared: normal, random, name appears after the first success (NAFS), and change to normal controller after the first success (CNFS). The normal layout roughly matched the position of the robot’s joints, whereas the random layout was randomly assigned, and no information was displayed on each button. Before completing the shift task, a random layout was provided in the NAFS and CNFS layouts. After the first success, the corresponding joint information was displayed in the NAFS layout, whereas the layout was changed to a normal one in the CNFS layout. In total, 51 participants used the normal layout, 7 participants used the random layout, 25 participants used the NAFS layout, and 24 participants used the CNFS layout. The results indicate that providing a random layout during the exploration process (before the first success) is preferable for effective exploration and learning. However, during the learning process (after the first success), providing the relationship between joint movements and buttons in a visual manner is better without changing the button layout from that used in the exploration process.

## Introduction

The demand for the usage of robots is increasing in several fields, including medical operations, welfare, housework, office work, and disaster response. Some medical robots are already at the practical level. These robots [1–7] are driven by operators to enhance the safety and accuracy of treatment, which, to date, cannot be guaranteed by artificial intelligence-based automation. In the near future, these types of robots that are remotely controlled by operators will be used in other fields, including homes, factories, and offices. Furthermore, the operators will include non-experts. Establishing a method for learning robot operations that can be easily mastered by non-experts is important. With this in mind, we aimed to develop a method that facilitates skill acquisition for non-experts that operate robots. As a first step, this study investigated the effect of input button layout on skill acquisition for non-experts.

There are two main methods for facilitating the acquisition of robot operation skills. One is to use simulators and the other is to develop operational devices that facilitate skill acquisition. Several simulation systems have been developed to operate medical robots [8–18]. The basic design concept of these simulators aims to resemble the original system, such that people who have acquired an operation skill in the simulation system can operate the actual system with minimal effort. Simulators can facilitate skill acquisition by providing many practice opportunities and longer practice times, even when actual robots are unavailable. However, simulators do not reduce the time required for acquiring the skill, and while some people can easily acquire this skill, others require more time. An investigation is required from the perspective of how quickly operation skills can be acquired.

Research on operational devices that facilitate skill acquisition can be divided into two types. One is to find or develop methods for increasing the intuitiveness of operational devices, and the other is to find or develop components of operational devices that facilitate skill acquisition.

Tablet-type input devices are widely employed and can be operated intuitively, even by non-expert users [19]. However, tablet devices provide a two-dimensional (2D) input space, whereas robots move in a three-dimensional space, and the interface elements used to control the robots can affect operation performance. Hashimoto et al. developed a system in which operators directly controlled an unmanned ground vehicle (UGV) manipulator by touching and moving a virtual manipulator on a tablet device. Although the developed system was intuitive, some participants requested a stylus pen for more precise control, whereas others said that the depth direction in the virtual space was not easy to understand [20]. Singh et al. developed a similar system in which an operator could intuitively select the robot motion trajectory through a drag-and-drop-like input operation [21]. This type of operation method can provide intuitive control of robots, but the advanced visualization and control algorithms involved result in high computational costs [22]. Efforts have been made to address these issues and reduce the computational burden [21, 23]. Several studies have attempted to reduce the difficulty of precision when typing on tablet devices. Bengel et al. developed an interface using a picture display [24]. Herbert et al. provided an interface that allowed only a high-level/meta-level input [25], wherein the low-level control was conducted autonomously. Suehiro et al. developed a 2D interface for realizing an assembly task by assigning a graphical assembly frame to predefined trajectories [26]. In summary, low-level controls were performed by systems, and high-level/meta-level controls were performed by human operators. The several control methods that can adapt unknown dynamics of robots [27, 28] can further improve the robustness in low-level controls. However, when the actual task is performed remotely from homes, offices, factories, and medical operating rooms, two main problems arise. One is the limitation of available motion trajectories, and the other is the difficulty in understanding the dynamics of an operating robot. In most systems, operators can use only predefined robot motions to perform the limited tasks. Even if the system can generate the motion trajectory for each unskilled operator’s request, a feasible motion trajectory may be impossible because the unskilled operator does not understand the dynamics of the operating robot, e.g., the needs to maintain body balance to avoid falling when operating multi-legged robots such as humanoid and quadruped robots and maintain grasps to avoid dropping the objects to be used in a task. In this case, generating a feasible motion trajectory according to the request may require a considerable amount of time. To perform actual work in the field using a teleoperated robot, non-experts must learn how the robot moves through operational experience, accounting for the effects of dynamics.

Examples of studies that have investigated the components of manipulators that facilitate skill acquisition are [29] and [30]. Lopez et al. compared the input elements for controlling manipulator movements, including button inputs, joystick inputs, touchscreen gestures, and tilt gestures, and revealed that button and joystick inputs provided a human operator more precise control [29]. Goldstain et al. evaluated interface components for teleoperating a robotic manipulator in terms of ease of learning, accuracy of operation, and method of operation [30]. Their results indicated that learning is easy when operating a robot by direct visual observation rather than by observation through a camera. Learning in a virtual environment before operating the actual robot is effective in shortening the learning time. They focused on the effects of components on skill learning at the meta-level (e.g., virtual environment vs. actual environment and camera-view observation vs. direct visual observation) but did not investigate the effect of low-level components (e.g., button and game controller layouts). To understand how robots move, considering the effects of dynamics, the effect of low-level components directly associated with the dynamics should be investigated.

To address these issues, we aimed to develop a method for reducing the time required for acquiring a robot operation skill, including understanding the robot motion under the influence of dynamics, and investigate the effect of input button layout on the learning process as a first step.

Typically, robots have numerous degrees of freedom (DOFs), and a corresponding number of commands is required for operation. Operators must select an appropriate sequence of commands among the commands available. The goal for the robot operation is given as a meta-goal, such as standing, sitting, or grasping. A corresponding para-goal is a sequence of commands that achieves the goal. There are multiple appropriate command sequences (para-goals), that is, there are multiple solutions and methodologies for achieving a single meta-goal. Therefore, an appropriate solution from many unspecified methodologies must be explored and learned to be able to repeat it. In the aforementioned studies, no investigation or approach was considered from this perspective. By contrast, the present study investigates the effect of input button layout on the exploration process.

A humanoid robot was selected as the target robot because it has a large number of DOFs, provides a dynamics consideration for its control (e.g., maintaining body balance to avoid falling down), and is unfamiliar to non-experts. Non-experts do not have the skill required to operate the robot; thus, the exploration and learning processes can be observed. The humanoid robot was operated using an unfamiliar tablet-based user interface to achieve the task of shifting the robot’s posture to the desired posture: single-foot standing. The process of participants finding and repeating command sequences to achieve the shift task was observed. Four types of button layouts were prepared: normal, random, name appears after the first success (NAFS), and change to normal controller after the first success (CNFS). The normal layout roughly matched the position of the robot’s joints, whereas the random layout was randomly assigned, and no information was displayed on each button. We expected the random layout to allow the participants to manipulate the robot without predicting how the robot’s state changed with the movement of the joint that they attempted to command. A complex button layout without displayed information can make remembering the specific buttons that generate a specific type of robot movement difficult. In particular, this disadvantage can become significant after a successful experience. To consider this effect, we prepared two additional types of layouts: NAFS and CNFS layouts. Before completing the shift task, a random layout was provided in the NAFS and CNFS layouts. After the first success, the corresponding joint information was displayed in the NAFS layout, whereas the layout was changed to a normal layout in the CNFS layout. The process before the shift task succeeds corresponds to the exploration process, and the process after the first success corresponds to the learning process. We compared the exploration and learning processes with the four layouts and investigated the effects of the input command layouts on them.

## Materials and methods

This section describes the participants, apparatus, procedure, and data analysis method.

### Participants

The present study was approved by the ethics committee of Kanazawa University. A total of 107 participants (51 females and 56 males; age: 20.7 ± 2.38 years) were recruited from Kanazawa University and related institutions (see Table 1 for details). All procedures involving human participants were conducted in accordance with the ethical standards of the institutional and national research committee and the 1964 Declaration of Helsinki, its later amendments, and comparable ethical standards. After receiving a complete explanation of the study, all the participants agreed to take part in the study and provided written informed consent.

**Table 1:** Participants who used normal, name appears after the first success (NAFS), change to normal controller after the first success (CNFS), and random controllers.

Used Controller	Normal	NAFS	CNDS	Random
**Female / Male**	22 / 29	13 / 12	13 / 11	3 / 4
**Age mean (SD)**	21.2 (1.799)	20.9 (1.72)	20.0 (3.69)	19.6 (1.40)

### Apparatus

**Fig 1:**
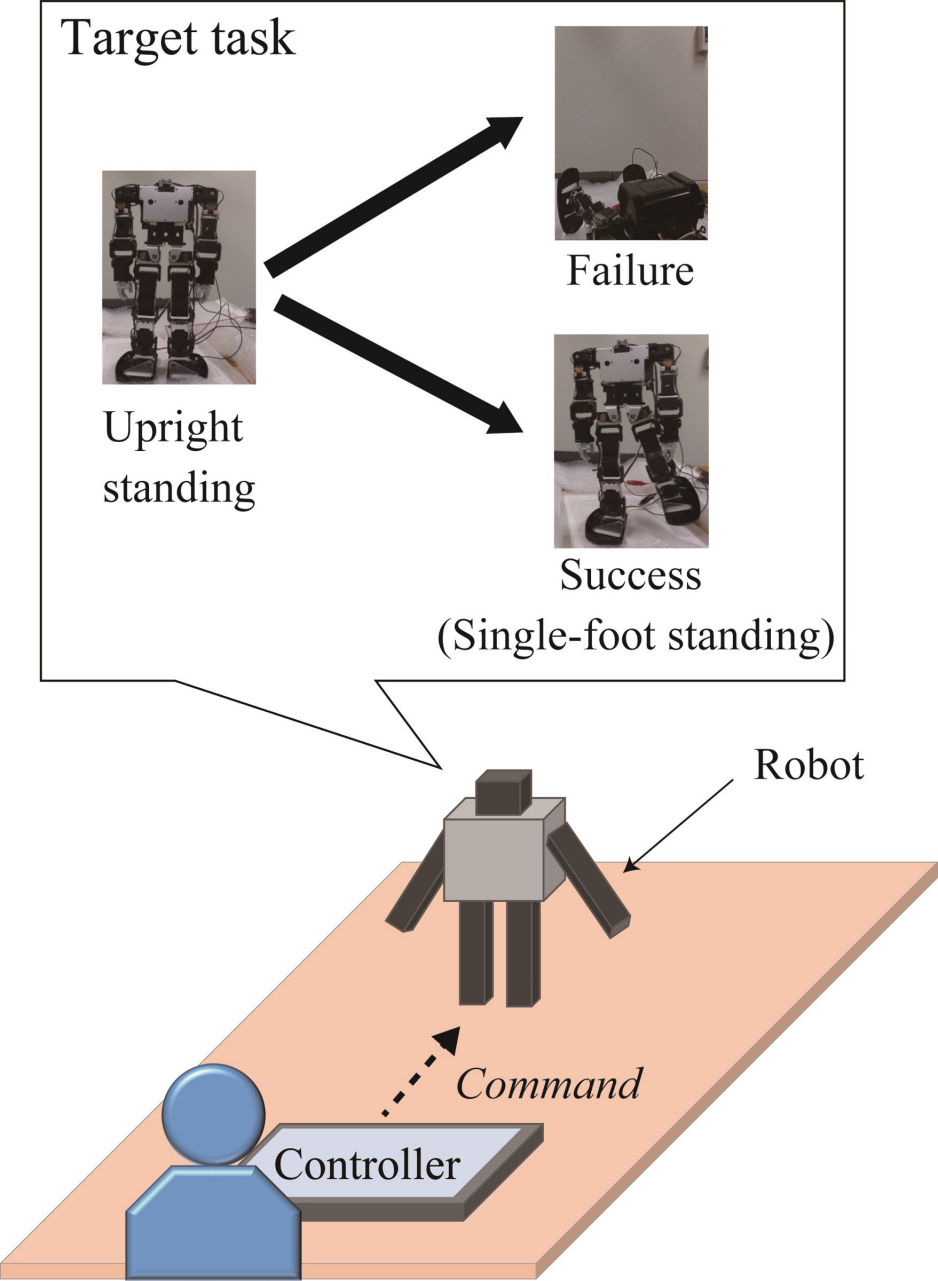
Overview of the target operation.

**Fig 2:**
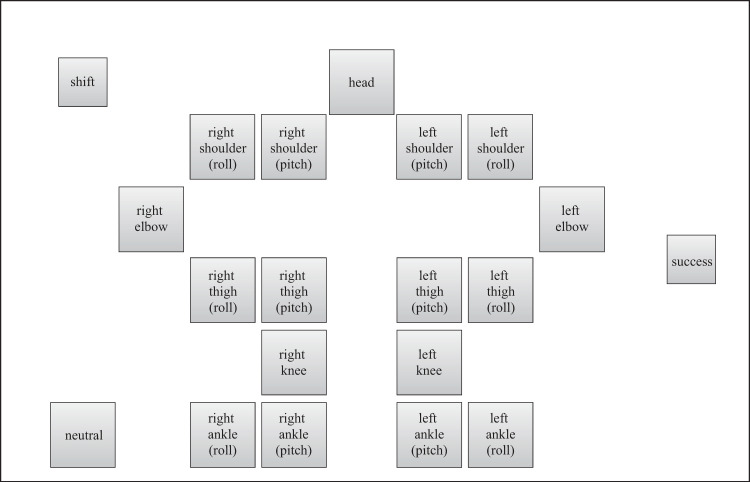
Command layout of the normal controller displayed on the tablet PC.

**Fig 3:**
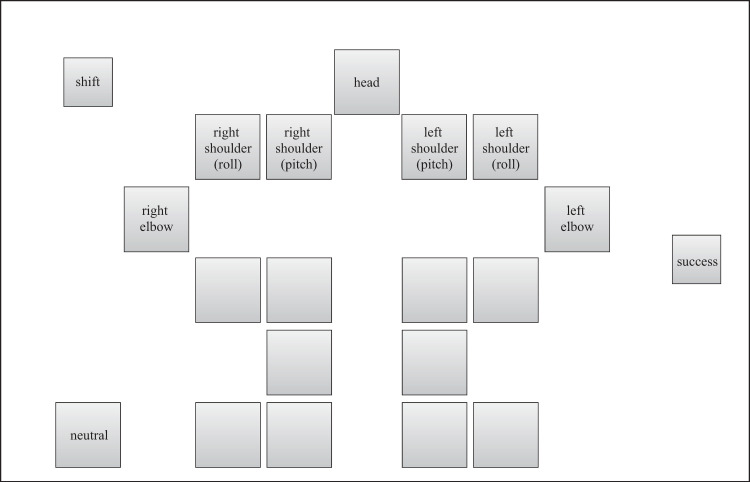
Command layout of the random controller displayed on the tablet PC.

**Fig 4:**
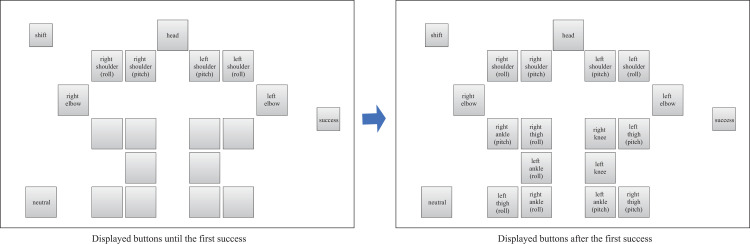
Command layout of the NAFS controller displayed on the tablet PC.

**Fig 5:**
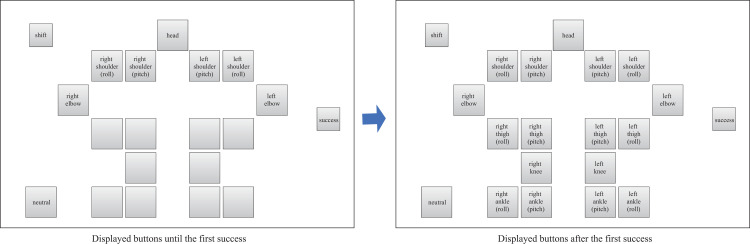
Command layout of the CNFS controller displayed on the tablet PC.

A Kondo Kagaku KHR-3HV ver. 2 was used as the robot to be controlled. We confirmed that this was the first time that all the participants had seen the robot and those who had seen it or experienced operating similar robots were excluded. As shown in Fig 1, the robot was controlled through a controller displayed on a tablet PC (Sony VAIO Duo 11). To observe the process of skill acquisition, we used a handmade controller that was unfamiliar to the participants (see Figs 2–5). The controller was written in Visual C#, based on the Rcb4 library provided by Kondo Kagaku, using Microsoft Visual Studio. When touching or pushing the button displayed on the touchscreen, the corresponding motor rotated at a constant angle of 3.13°.

The four types of controllers shown in Figs 2–5 were prepared to examine the effect of the input button layout on the exploration and learning processes for robot operations. In the normal controller (Fig 2), the location of the input buttons roughly matched the position of the joints of the robot. Each button corresponded to a joint, except for the shift, neutral, and success buttons. If the shift button was touched, the rotational direction changed from positive (negative) to negative (positive). If the neutral button was touched, the robot automatically moved to the initial upright standing posture. If a trial was successful, the success button was to be touched. If the buttons for joint motions were touched, the corresponding number and time it was touched were recorded for analysis. A preliminary experiment revealed that at least 1 h was required, even for experts, if a participant attempted to control all the joints. Therefore, all the buttons related to the movement of the upper body were made unresponsive such that only the lower body could move. In contrast, in the random controller (Fig 3), the location of the input buttons did not match the position of the robot’s joints. Additionally, no information was displayed on the buttons to prevent the participants from knowing which button moved a particular joint. As mentioned above, we expected this setting to allow the participants to manipulate the robot without predicting how the robot would move according to their commands. A complex button layout can make remembering the buttons commanded difficult. Therefore, a user may want to repeat a successful command sequence after success, but this can be difficult. To consider this effect, we prepared two different types of controllers: NAFS (Fig 4) and CNFS (Fig 5). Until the first success, these controllers were the same as those of the random controller. After the first success, the information about which joint was moved was displayed on the button in the NAFS controller, whereas the random controller was replaced by the normal controller in the CNFS controller. We examined which change benefited the participants.

### Procedure

As shown in Fig 1, the operation task was to shift the posture from the initial upright posture to the single-foot standing posture. The single-foot standing posture is defined as standing with the left foot off the ground without falling under steady-state conditions. After the explanation of the task mentioned above, the operation method of the normal controller was instructed to the participants while demonstrating how the robot moved using the commands provided. The relationship between the buttons, joints, and their rotational directions was also examined by the participants. After receiving instructions, the participants operated the robot. Each trial started from the robot in the initial upright standing posture, and participants attempted to have it attain a single-foot standing posture. If the participants wanted to restart the task (for example, when the robot fell down) during each trial, they were instructed to touch the “neutral” button. If the participants succeeded in shifting to a single-foot standing posture, they were instructed to touch the “success” button after the trial.

The terminal condition for the task was set to have three consecutive successes within 1 min without giving up a trial or making the robot fall. The reason we put a time limit on success was to exclude successes that involved excessive trial and error. We judged that the participants reached a certain level of understanding of robot operation by observing the conditions of their success. The number of repeat trials and time limit for success were defined based on the results of preliminary studies. Note that the experiment was also stopped if 1 h had elapsed without completing the terminal condition, accounting for the effects of fatigue and declining concentration.

### Data analysis

We observed two types of abilities in the participants. One was how quickly they could find unspecified and appropriate command sequences to complete the desired posture. The other was how fast they could produce the appropriate command sequence repeatedly, that is, how fast they could learn the appropriate command sequence.

MATLAB (MathWorks) was used to perform statistical analyses. The descriptive statistics of the samples were calculated. The differences in the time and number of commands required for the first success, total operation time, and total number of commands between the groups were analyzed using a one-sided Wilcoxon rank-sum test to evaluate which group had a larger value. If a participant did not succeed in the task (not even once), the number of commands and duration required for the first success in the task were recorded as the maximum values: the total number of commands and 60 min, respectively. To evaluate the differences in these analyses, the significance level was set at 0.05. Additionally, to determine the effect of the random layout of command buttons and evaluate how much the same type of commands were used, the ratio of the top-three commands used to the total number of commands used to achieve the first success and terminal condition was derived. “Three” was set such that the maximum ratio was close to 1 when using a normal controller (the ratio for at least one participant was close to 1). To observe the effect of the random layout after the first success, we also evaluated the ratio of the success trials from the first success until the terminal condition was met.

## Results

**Fig 6:**
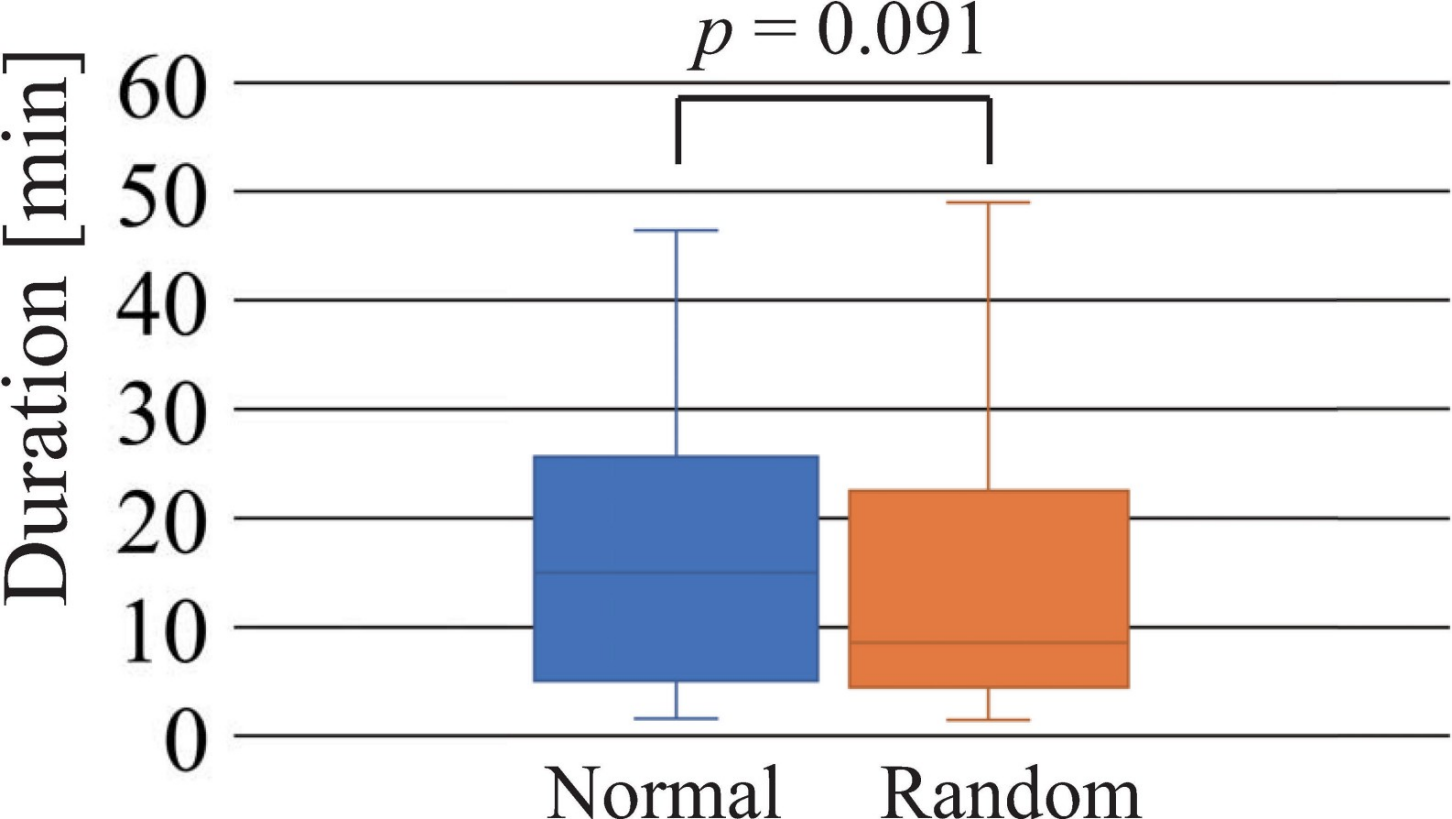
Duration required to achieve the first success of the shift task. Normal and random indicate the normal and random controllers, respectively.

**Fig 7:**
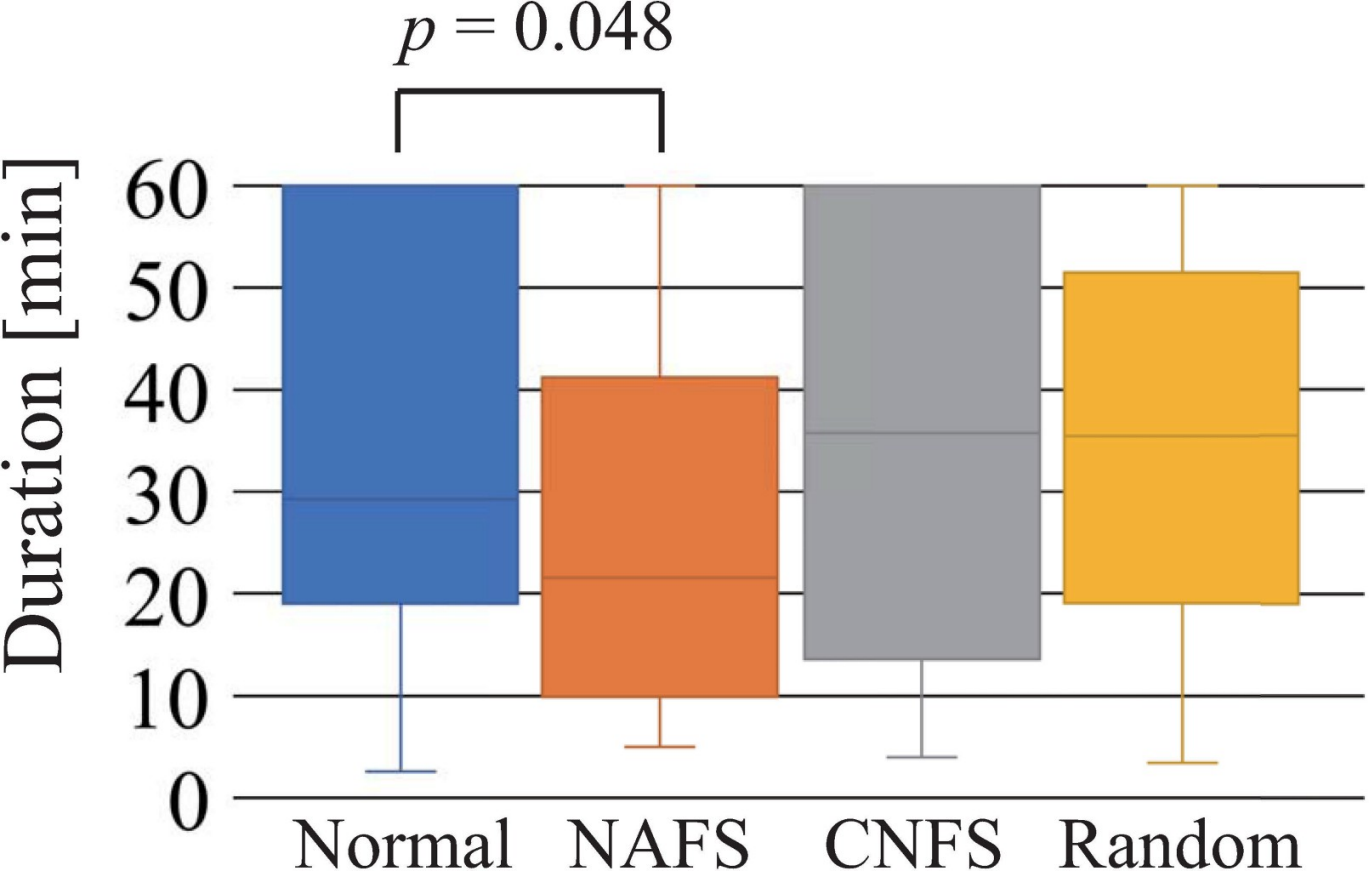
Duration required to achieve the terminal condition. This indicates a certain level of understanding of the operation. Normal, NAFS, CNFS, and random indicate the normal, “name appears after the first success,” “change to normal controller after the first success,” and random controllers, respectively.

This section shows the results for duration, number of commands, ratio of the top-three commands used, and ratio of success trials from first success until the terminal condition was met. Fig 6 shows the duration required to achieve the first success of the shift task. Because the NAFS and CNFS controllers are equal to the random controller until the first success, the result for the random controller includes the results of the NAFS and CNFS controllers. Although a statistically significant difference between the normal and random controllers was not observed, the participants using the random controller tended to find a successful motion sequence more quickly than the participants using the normal controller. Fig 7 shows the duration required to achieve the terminal condition. A statistically significant difference between the normal and NAFS controllers indicates that the NAFS controller facilitated the skill acquisition of robot operation (to a certain level of understanding of the operation). No distinctive differences were observed between the normal and random controllers or between the normal and CAFS controllers.

**Fig 8:**
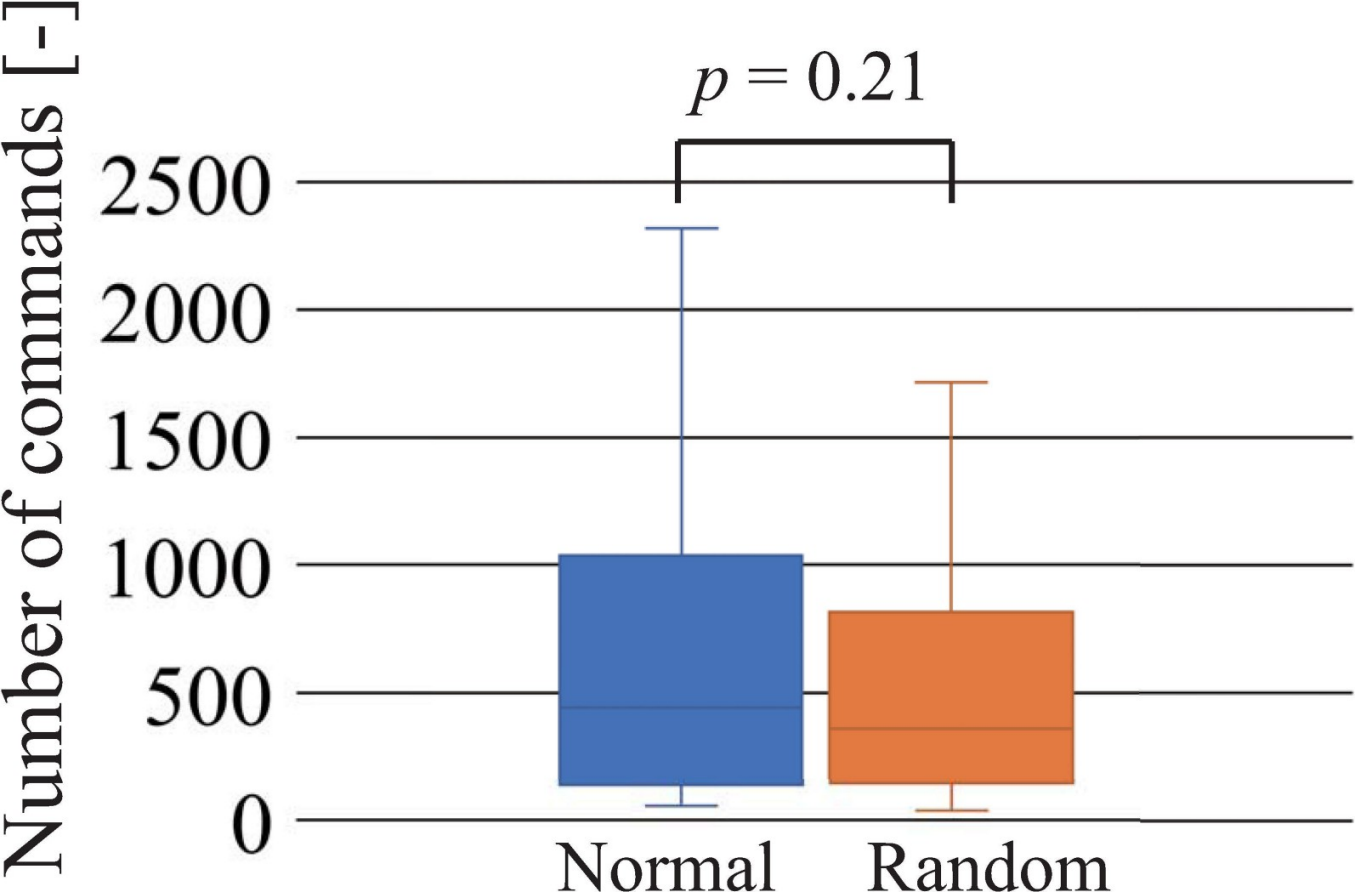
Number of commands required to achieve the first success of the shift task. Normal and random indicate the normal and random controllers, respectively.

**Fig 9:**
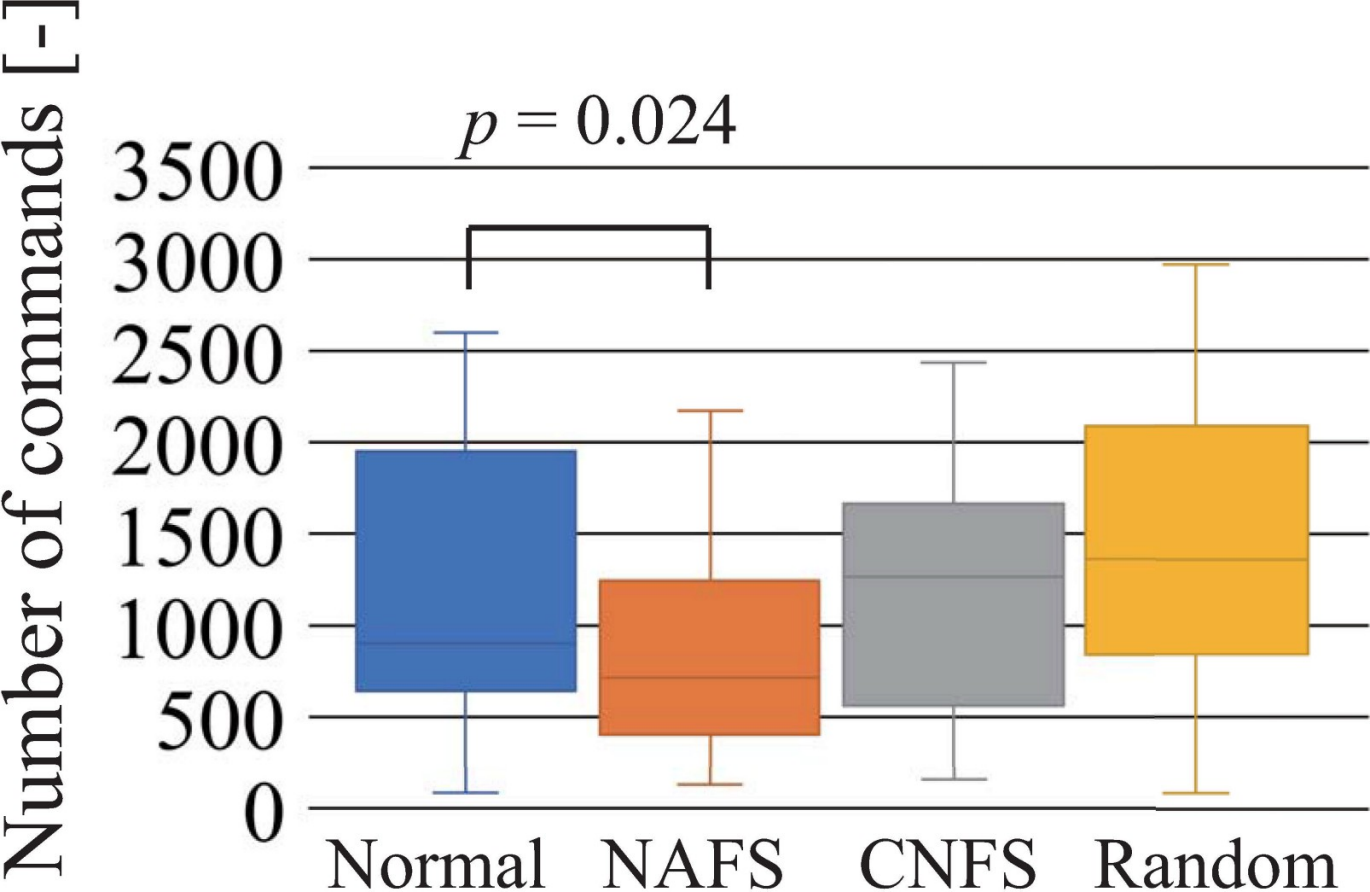
Number of commands required to achieve the terminal condition. This indicates a certain level of understanding of the operation. Normal, NAFS, CNFS, and random indicate the normal, “name appears after the first success,” “change to normal controller after the first success,” and random controllers, respectively.

Fig 8 shows the number of commands required to achieve the first success of the shift task. Similar to the required duration, participants using the random controller tended to find a successful motion sequence more quickly than those using the normal controller, although a statistically significant difference between the normal and random controllers was not observed. Fig 9 shows the number of commands required to achieve terminal conditions. Similar to the required duration, a statistically significant difference was observed between the normal and NAFS controllers.

**Fig 10:**
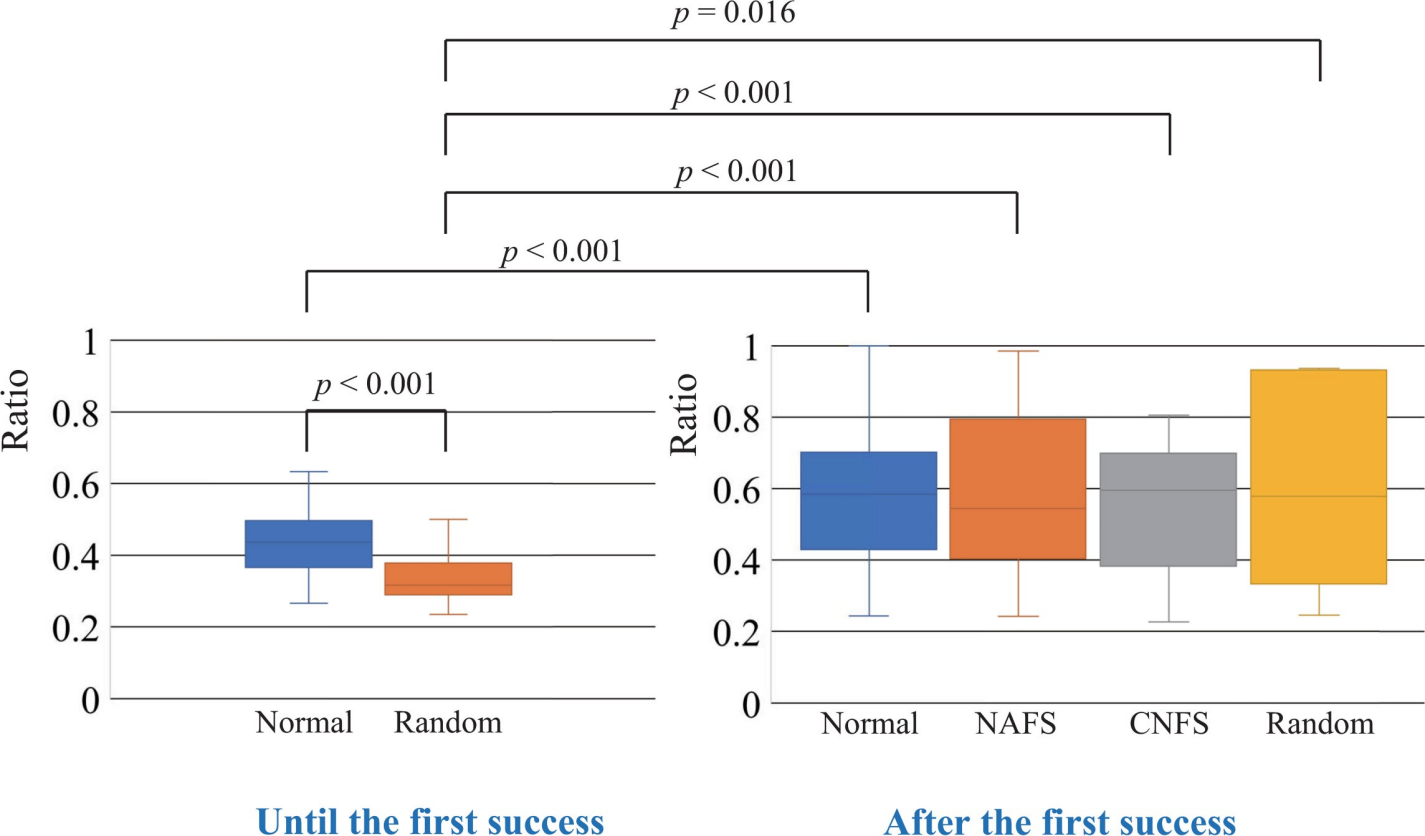
Ratio of the top-three commands used to the total number of commands used. The left part of the figure shows the ratio for achieving the first success of the shift task, and the right part shows the ratio from the first success to the timing when achieving the terminal condition.

Fig 10 shows the ratio of the top-three commands used to the total number of commands used to determine the effect of layout. The left part of the figure shows the ratio for achieving the first success of the shift task, and the right part shows the ratio from the first success until the terminal condition was met. Until the first success, the ratio of using the normal controller was significantly larger than that using the random controller. After the first success, the ratio significantly increased for each controller, as shown in Fig 10 (right). Note that because the NAFS and CNFS controllers are equal to the random controller until the first success, we compared the random controller until the first success with the NAFS and CNFS controllers after the first success. No distinctive differences were observed between the normal, NAFS, CNFS, and random controllers after the first success.

**Fig 11:**
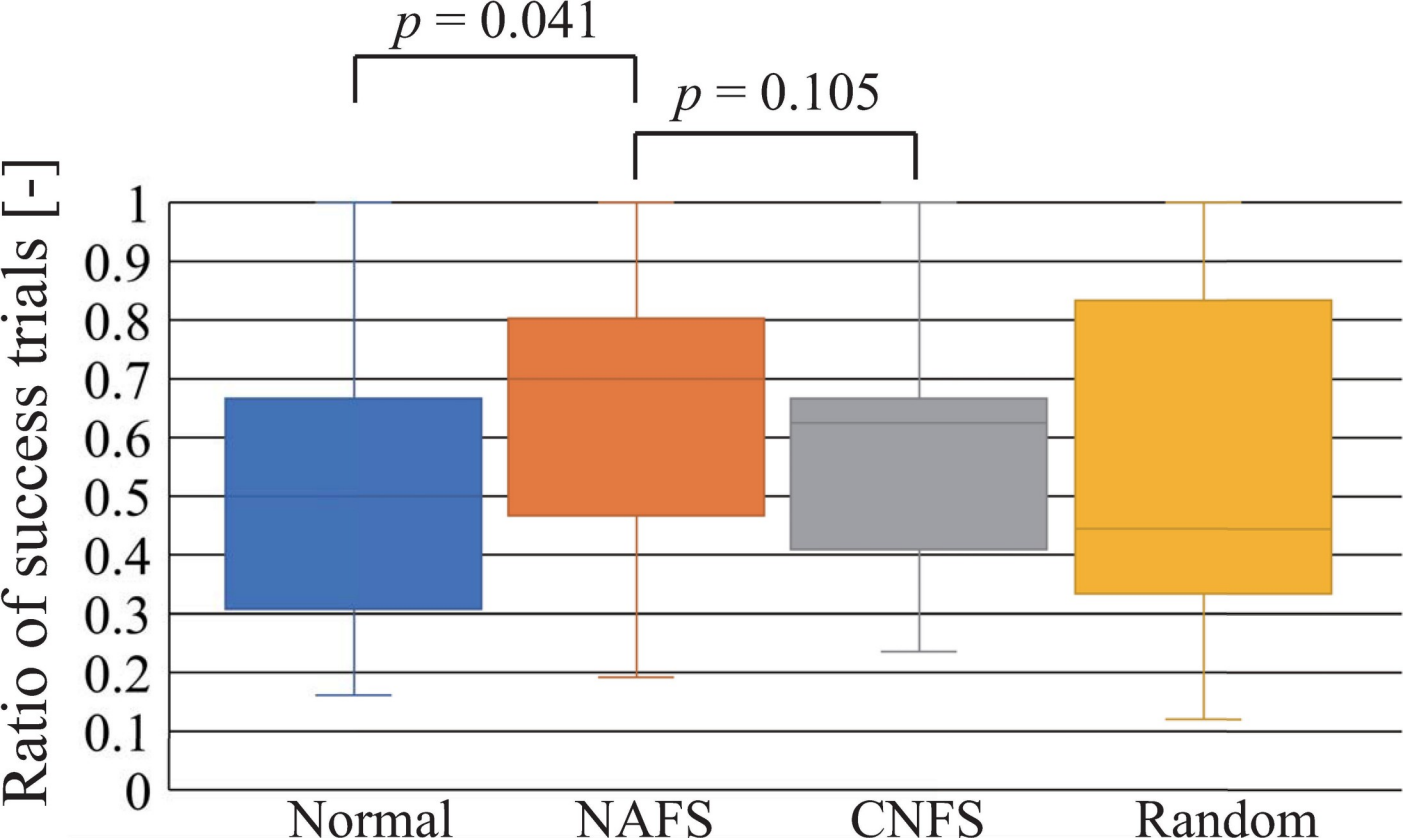
Ratio of success trials for achieving the terminal condition.

Fig 11 shows the ratio of success trials from the first success until the terminal condition was met to examine the effect of layout after the first success. The participants using the NAFS controller could generate a command sequence with a high success rate. In particular, the success rate was significantly higher than that when using the normal controller.

## Discussion

This section first discusses the results obtained, followed by their limitations. Among the four controllers, the NAFS controller provided the fastest exploration and learning in terms of the duration and number of commands required for achieving the terminal condition, particularly when compared to the normal controller. Because the solution was unknown until the first success, the behavior until the first success can be viewed as an exploration process. After the first success, the process shifts to a learning process, where it becomes important to determine how efficiently the obtained solution can be repeated. The obtained results indicate that the random layout of the command buttons is preferable during the exploration process. However, during the learning process, it is preferable to provide the relationship between joint movements and buttons visually without changing the button layout from that used in the exploration process. During the exploration process or until the first success, the random layout of the command buttons allows efficient exploration of the solution, although the effect of the random layout is insignificant, as shown in Figs 6 and 8. The results shown in Fig 10 indicate that until the first success (in the exploration process), participants with a random controller used more types of commands to explore a solution than participants with a normal controller. In other words, the exploration range when using a random controller was larger than that when using a normal controller. This might be the reason why the random controller was effective in the exploration process.

In the NAFS controller, the randomly-assigned layout of the command buttons did not change even after the first success (during the learning process), but the relationship between the joint movement and buttons was visualized. Therefore, the participants continued to operate the robot with a non-intuitive button layout. Because it was difficult for them to find other solutions (other command sequences for achieving a single-foot standing posture), it is likely that they tried to repeat the command sequence obtained in the first success as much as possible. The high success ratio, shown in Fig 11, and the high ratio of the top-three commands used, presented in Fig 10, support this inference. This result is contrary to what has been considered a good, more intuitive user interface [19–26, 29].

In the CNFS controller, the button layout changed to the normal layout after the first success. Although the normal layout was more intuitive than the random layout, it is believed that it is necessary to remap the relationship between the commands and their locations to generate the command sequence obtained in the first success (or find other successful command sequences); thus, it takes time to learn the operation owing to the difference in layout. This is supported by the fact that the ratio of success trials and the range shown in Fig 11 are close to those obtained using the normal controller. Because the normal controller is intuitive, the first success in the normal controller did not produce a big surprise or impact compared to the first success in the random controller. Surprises or impacts create a strong memory [31–33]. The command sequence of the first success with the normal controller is slightly vaguer in memory than that of the random controller. Additionally, some participants were willing to continue exploring other types of solutions according to their comments after the experience. This could be the reason why the success rate of the normal controller was lower than that of the NAFS controller. These factors could also affect the duration and number of commands required for termination.

As shown in Fig 10, the results indicate that after the first success (in the learning process), the participants tended to try to use more of the same type of commands with any controller than they did before they succeeded the first time. If the correct command sequence can be approximately estimated, it is easier to find the correct solution quickly by searching around it. This method is called a local search [34]. However, if the estimation is incorrect, it is difficult to reach the correct solution. In this case, it is efficient to explore the solution randomly without estimation. This is called a random search [34]. At the first success, participants knew the correct command sequence. This allowed them to search for the correct command sequence or repeat it if they remembered the correct command sequence. The behavior after the first success shown in Fig 10 corresponds to a local search. In contrast, the behavior up to the first success in the case of using the random controller corresponded to a random search. The appearance of the humanoid robot used was similar to that of humans, whereas its dynamics were different from those of humans. Therefore, it was easy to fail if a participant thought that the robot should be able to shift to a single-foot standing posture if they operated the robot in the same manner as humans. Some participants said that they tried to move the robot like their own body, but it did not work. This might be a reason why the exploration range until the first success was too small to perform an efficient random search when using the normal controller.

The investigated method can be applied to the case in which a beginner starts learning a robot operation. For example, the learning system can be configured as follows. The input commands are randomly placed without displaying command information until the first simple task succeeds. After the first success, command information is displayed. This method can be applied to interfaces beyond tablet types. Furthermore, the control target is not limited to humanoid robots; other robots, vehicles, and machines can also be control targets. Further investigation is required to determine whether this method is effective for other user interfaces and targets.

### Limitation

When using the random controller, the behavior after the first success varied significantly, as can be observed from the large range in the ratio of the top-three commands used (Fig 10) and ratio of successful trials (Fig 11). Therefore, we focused on layout changes after the first success, and the number of participants using the random controller was low. The target participants were those unfamiliar with robot controls; thus, the results could change if the participants were familiar with robot control. This study focused on the difference between the normal and random layouts of command buttons, to observe the effect of extending the exploration range using a random layout on exploration and learning. The random layout might have encouraged participants to perform random search. In this sense, the random layout design is based on random search. There are more sophisticated search algorithms [35–38], and layouts can be designed based on these search algorithms. Therefore, the optimal layout among the targeted layouts was obtained, and more efficient layouts could exist. The system provided a simple control in which robot joints moved according to input commands. This control method is effective for non-experts to learn how the robot moves through operational experience, accounting for the effects of dynamics. In contrast, conducting complex tasks using this control method is difficult because the control cost for operators is high. More sophisticated control methods [27, 28] are required to conduct complex tasks and reduce the control cost for non-experts. Note that even when the control cost is reduced by a controller, non-experts should know how the robot moves—in consideration of the effects of dynamics—to understand the effect of robot motion on the surroundings and the robot itself. Therefore, non-experts must learn robot operation in a step-by-step manner, increasing the complexity of the task and raising the level of the control method accordingly. The type of controller appropriate for each task level should be considered simultaneously. The relationship between the learner’s level of understanding and level of the applied controller is unclear. The challenge of tackling these issues will be addressed in future work. Only a tablet-type controller and humanoid robots were considered, and the target robot was operated while directly viewing it. The investigation of other types of robots with other types of controllers, including game pads, and investigations in other environments, such as virtual ones, will also be involved in future work.

## Conclusion

This study investigated the effect of the command button layout on the exploration and learning processes of humanoid-robot operation. We observed a process in which participants unfamiliar with robot operations used variously placed input buttons to find and repeat sequences of commands to shift the robot’s posture to a desired posture (single-foot-standing). The obtained results indicate that providing a random layout of command buttons is preferable for effective exploration and learning during the exploration process (before the first success of the shifting task). However, during the learning process (after the first success), providing the relationship between joint movements and buttons in a visual manner, without changing the button layout from that used in the exploration process, is better.

## Supporting information

S1 Data (XLSX)
